# Predictive Value of Procalcitonin for Bacterial Infection after Transarterial Chemoembolization or Radiofrequency Ablation for Hepatocellular Carcinoma

**DOI:** 10.1155/2018/9120878

**Published:** 2018-04-17

**Authors:** Seung Ji Kang, Uh Jin Kim, Seong Eun Kim, Joon Hwan An, Mi Ok Jang, Dae-Seong Myung, Kyung-Hwa Park, Sook-In Jung, Sung Bum Cho, Hee-Chang Jang, Young Eun Joo

**Affiliations:** ^1^Division of Infectious Diseases, Department of Internal Medicine, Chonnam National University Medical School, Gwangju, Republic of Korea; ^2^Department of Internal Medicine, Mokpo Hankook Hospital, Mokpo, Jeollanam-do, Republic of Korea; ^3^Department of Internal Medicine, Presbyterian Medical Center, Jeonju, Jeollabuk-do, Republic of Korea; ^4^Division of Gastroenterology, Department of Internal Medicine, Chonnam National University Medical School, Gwangju, Republic of Korea

## Abstract

This prospective observational study aimed at investigating the role of procalcitonin (PCT) in diagnosing bacterial infection and guiding antibiotic therapy for hepatocellular carcinoma (HCC) patients with fever after transarterial chemoembolization (TACE) and/or radiofrequency ablation (RFA). Ninety-seven cases (84 patients) were enrolled. Serum PCT, C-reactive protein (CRP), and white blood cell (WBC) counts were measured on the day of fever onset (day 0) and days 1, 3, 5, and 7 of fever. Empirical antibiotics were initiated only if PCT was ≥0.5 ng/mL or specific infection foci were suspected. An infectious cause was found in nine cases. PCT on day 0 of fever was significantly higher in patients with bacterial infection than in those without infection (*P* = 0.035). The area under the receiver operating characteristic curve for PCT was 0.715 (95% confidence interval, 0.538–0.892) and was higher than that for CRP (0.598 (0.368–0.828)) or WBC counts (0.502 (0.307–0.697)). In patients undergoing TACE and/or RFA, a significantly lower number of antibiotics were prescribed during the study period than during the prestudy period (*P* < 0.001). In conclusion, PCT might be a biomarker for diagnosing infection and guiding antibiotic treatment to reduce unnecessary antibiotic use in patients with fever after TACE and/or RFA.

## 1. Introduction

Transarterial chemoembolization (TACE) and radiofrequency ablation (RFA) are the most widely used primary treatments for unresectable hepatocellular carcinoma (HCC) [1]. These procedures are generally well tolerated with a low rate of major complications (4–7% in TACE and 2-3% in RFA) such as acute hepatic failure, liver infarction, cholecystitis, intrahepatic biloma, and hepatic abscess [2–4]. However, up to 74% or 35% of patients undergoing TACE or RFA, respectively, experience temporary fever, abdominal pain, and related constitutional symptoms defined as postembolization or postablation syndrome [5, 6]. The pathogenesis of postembolization or postablation syndrome remains unclear. In most patients, the appearance of fever after these procedures represents extensive necrosis of the tumor and healthy tissue [7]. Infectious complications are very rare for both of these procedures [4, 5]. Whatever the etiology might be, fever causes concern and frequently leads physicians to prescribe unnecessary antibiotics. The concern of physicians is usually based on the fact that baseline cirrhosis of HCC patients is an independent risk factor for sepsis and a poor outcome of infectious complications [8, 9].

Procalcitonin (PCT) is a 116-amino-acid prohormone of calcitonin, which is normally synthesized in C cells of the thyroid gland. The diagnostic value of PCT to predict bacterial infection has been studied in various diseases and patient groups [10, 11]. In patients with decompensated liver cirrhosis, PCT showed the best diagnostic value relative to C-reactive protein (CRP), interleukin-6, and tumor necrosis factor-*α* [12]. Dabbous et al. reported the clinical usefulness of PCT for diagnosing bacterial infection in postembolization syndrome [13]. However, their study was performed in a relatively small number of patients, and the clinical application of PCT on the decision to start antibiotic treatment has never been validated. This study aimed at investigating whether PCT could be adopted as a biological marker for diagnosing bacterial infection and guiding antibiotic therapy in patients with fever after TACE and/or RFA to treat HCC.

## 2. Patients and Methods

### 2.1. Study Population

This prospective study was performed between December 2012 and December 2013 in Chonnam National University Hwasun Hospital (Jeonnam, Korea). All patients admitted for TACE and/or RFA to treat HCC were screened during the study period. Patients who developed fever (≥38.0°C) within 7 days after TACE and/or RFA were included. Patients with documented fever, infection, or antibiotic use within 7 days before TACE and/or RFA were excluded. To compare the amount of antibiotic use between the study period (December 2012 through December 2013) and the prestudy period (November 2011 through November 2012), all medical records of patients admitted for TACE and/or RFA in the prestudy period were retrospectively reviewed. This study was approved by the institutional review board of Chonnam National University Hwasun Hospital (approval number 2012-190). Written informed consent was obtained from all participants before enrollment in this study.

### 2.2. Disinfection Process before and during TACE and/or RFA

Preprocedural care for TACE was performed including hydration, antiemetics and antihistamine, and a single dose of dexamethasone 5 mg. Although some recommend the prophylactic antibiotics for TACE in patients with severe liver cirrhosis, none of the patients included in this study received the prophylactic antibiotics, because their Child-Pugh score is A or B [14, 15]. During the procedure, for skin disinfection, chlorhexidine-based solution (>0.5% chlorhexidine preparation with alcohol) was used. Sterile techniques including hand washing, aseptic technique, use of sterile gloves, long-sleeved surgical gown, a surgical mask, and a large sterile sheet drape were applied.

### 2.3. Study Design and Intervention

PCT, CRP, white blood cell (WBC) counts, total bilirubin, albumin, alkaline phosphatase (ALP), aspartate aminotransferase, alanine transaminase, prothrombin time (international normalized ratio), blood urea nitrogen, creatinine, and electrolytes were measured just before TACE or RFA and on the day of fever onset (day 0). PCT was measured using an immunoluminometric assay (Elecsys Brahms PCT; Roche, Germany) and the Cobas e602 analyzer (Roche) with a lower detection limit of 0.02 ng/dL. On day 0 of fever, two sets of blood culture samples were collected, and urinalysis and chest radiography were performed for all patients to evaluate the possible infection foci. Other imaging studies, ascites analysis, or culture of clinical samples such as sputum, urine, ascites or pus, and additional blood cultures were performed if indicated. An attending physician and infectious disease specialist examined each patient separately to evaluate the infection foci daily for 7 days after fever developed. We used the most widely used cutoff value for PCT of 0.5 ng/mL for the diagnosis of bacterial infection [16–18]. If the PCT level was <0.5 ng/mL and no specific infection foci were observed, no antibiotic was prescribed. However, if the PCT level was ≥0.5 ng/mL or specific infection foci were suspected, empirical antibiotic therapy, mostly third-generation cephalosporin with or without metronidazole, was initiated. The initial empirical antibiotics were switched to definitive antibiotics according to culture results or discontinued if no further evidence of bacterial infection was observed. All bacterial infections that occurred within the 7 days after TACE and/or RFA were included in this study. Serum PCT, CRP, and complete blood cell counts were also measured 1, 3, 5, and 7 days after fever developed. These measurements were discontinued upon discharge for patients who recovered and were discharged before 7 days after fever developed. Clinical data including age, sex, medical history, etiology of liver cirrhosis, presence of esophageal varices, ascites, and history of hepatic encephalopathy were collected.

### 2.4. Definition

The diagnosis of HCC was confirmed either by histological examination or by the clinical diagnosis: (1) chronic viral hepatitis infection or liver cirrhosis and, if the serum *α*-fetoprotein (AFP) level is ≥200 ng/mL, typical characteristic findings of HCC on dynamic contrast enhancement computed tomography (CT) or magnetic resonance imaging (MRI) or, if the serum AFP level is <200 ng/mL, typical characteristic findings of HCC in 2 or more radiologic studies (dynamic contrast enhancement CT or MRI or hepatic artery angiography), or (2) liver cirrhosis with a tumor ≥ 2 cm and typical characteristic findings of HCC on dynamic contrast enhancement CT or MRI regardless of serum AFP level. Typical characteristic findings of HCC are defined as contrast enhancement in the arterial phase and washout in the portal/venous phase in comparison with liver parenchyma [19, 20]. TNM staging was determined according to the cancer staging manual of the American Joint Committee on Cancer [21]. Fever after TACE and/or RFA was defined as a body temperature ≥ 38.0°C within 7 days of the procedure [7]. Infection was diagnosed if microbiological cultures obtained from the patients at possible sites of infection were positive or if clinical signs or imaging results showed evidence of infection. Primary bacteremia was considered when clinical symptoms and signs of infection were present and confirmed by microbiological demonstration of the causative organism in a blood culture sample in the absence of site-specific infection.

### 2.5. Statistical Analysis

Discrete variables are expressed as frequency (percentage) and continuous variables as means and standard deviations or median and interquartile ranges (IQRs) according to their homogeneity and distribution. Fisher's exact test or the Pearson *χ*^2^ test as appropriate was used for comparing categorical variables. Continuous variables were analyzed using the Mann–Whitney *U* test. To compare the predictive value of CRP, PCT, WBC counts, and neutrophil percentage, receiver operating characteristic (ROC) curves were constructed and the area under the curve (AUC) was determined. The outcome variable was the development of bacterial infection during the first week after TACE and/or RFA. All tests of significance were two-tailed, and *P* values < 0.05 were considered statistically significant. The Statistical Package for the Social Sciences (SPSS/PC +18.0, Chicago, IL) was used for statistical analyses and graphics.

## 3. Results

### 3.1. Basal Characteristics of Patients with Fever after TACE and/or RFA

Of the 563 TACE and/or RFA procedures performed during the study period, 97 cases (84 patients) of fever after TACE and/or RFA were included in this study. An infectious cause was found in nine (9.3%) of these cases, but no infectious cause was found in the other 88 cases (90.7%). The clinical characteristics and status of HCC showed no statistical differences between the two groups (Table 1).

The mean duration of fever was 1.2 days ranging from 1 to 8 days. For each patient with or without bacterial infection, the median duration of fever was 1.0 day (ranging from 1 to 8 days) and 1.0 (ranging from 1 to 6 days). Most of the patients (94 of 97 patients) developed the fever within 3 days after TACE and/or RFA. The overall median of peak body temperature was 38.1°C ranging from 38.0 to 39.8. For each patient with or without bacterial infection, the median of peak body temperature was 38.0°C (ranging from 38.0 to 39.8) and 38.1°C (ranging from 38.0 to 39.2). There was no statistical difference in peak body temperature and duration of fever between patients with and without bacterial infection.

Antibiotics were prescribed in 25 cases (9 of 9 patients with bacterial infection and 16 of 88 patients without the evidence of bacterial infection on further examination). The median duration of antibiotic therapy was 8.0 days (IQR 4.5–13.0), 13.0 days (IQR 5–18) in patients with bacterial infection, and 6.0 days (IQR 2–10) in patients without bacterial infection.

### 3.2. Clinical Characteristics of Nine Patients with Bacterial Infection after TACE and/or RFA

Of the nine cases of proven bacterial infection, hepatobiliary infection such as cholecystitis, liver abscess, or cholangitis was the most common (three cases) followed by urinary tract infection (UTI) and pneumonia (two cases each) and RFA site cutaneous abscess and bacteremia with unknown primary focus (one case each) (Table 2).

### 3.3. Association of Laboratory Markers and Bacterial Infection after TACE and/or RFA

Serum PCT levels measured on day 0 of fever were significantly higher in patients with bacterial infection (median 0.2 ng/dL, IQR 0.2–1.4) than in those without infectious etiology (median 0.1 ng/dL, IQR 0.1–0.3) (*P* = 0.035). However, there were no significant differences in WBC counts, neutrophil percentage, or CRP level between patients with and without bacterial infection. ALP and total bilirubin levels were also higher in patients with bacterial infection than in those without infection (*P* = 0.038 and *P* = 0.009, resp.) (Table 3).

### 3.4. Comparison of the Biomarker Kinetics between Patients with and without Bacterial Infection among Those Who Had Fever after TACE and/or RFA

**Figure 1:**
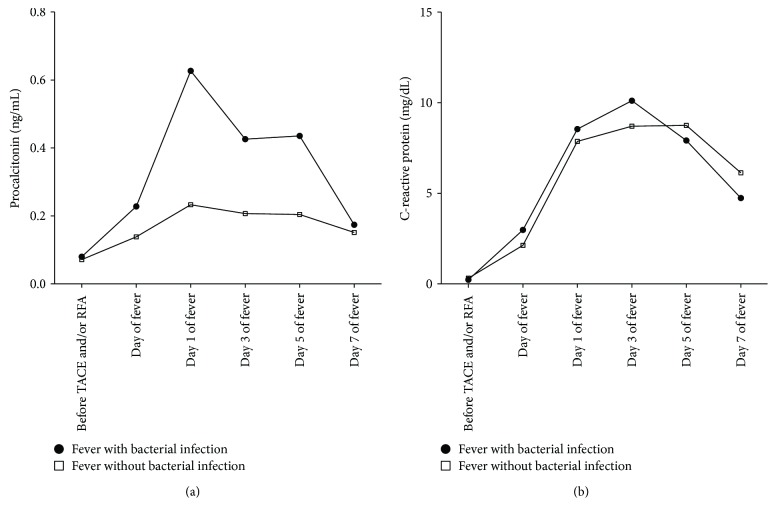
Median procalcitonin (a) and C-reactive protein (b) values over 7 days after onset of fever according to the etiology of fever. Basal, afebrile status before TACE or RFA.

The kinetics of PCT and CRP over 7 days after fever are shown in Figure 1. The peak value of PCT was observed on day 1 after the onset of fever in both patients with or without bacterial infection. On the other hand, highest CRP was observed on day 3 of fever in patients with bacterial infection and on day 5 of fever in those without bacterial infection. The median PCT level was significantly higher in patients with bacterial infection than in those without infection through days 0 to 5 of fever (*P* < 0.05), but there was no significant difference at 7 days of fever (median ng/dL (IQR), 0.17 (0.16 ~ 0.38) versus 0.15 (0.12–0.93), *P* = 0.556). There was no significant difference in the median CRP level between the two groups.

### 3.5. Comparison of Area under ROC Curves for CRP, PCT, WBC Counts, and Neutrophil Percentage in the Prediction of Infection in Patients with Fever after TACE and/or RFA

**Figure 2:**
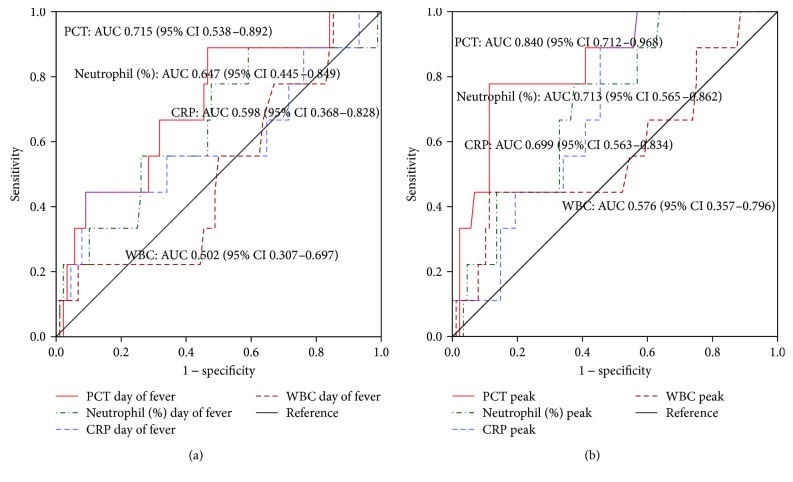
Receiver operating characteristic (ROC) curves and area under the curve (AUC) determined to predict the bacterial infection in patients with fever after TACE and/or RFA based on day of fever (a) and peak (within 7days of fever) (b) level of PCT (procalcitonin), CRP (C-reactive protein), WBC (white blood cells) counts, and neutrophil percentage.

The AUC (95% confidence interval (CI)) of PCT on day 0 of fever was 0.715 (0.538–0.892), which was higher than that of CRP (0.598 (0.368–0.828)), WBC counts (0.502 (0.307–0.697)), and neutrophil percentage (0.647 (0.445–0.849)) (Figure 2(a)). When the ROC curves were constructed based on the peak value of inflammatory markers, the AUC (95% CI) of peak PCT was 0.840 (0.712–0.968), whereas other inflammatory markers showed a relatively lower accuracy to predict the bacterial infection (CRP: 0.699 (0.563–0.834); WBC counts: 0.576 (0.357–0.796); neutrophil percentage: 0.713 (0.565–0.862)) than PCT (Figure 2(b)).

### 3.6. Comparison of Antibiotic Use in Patients with Fever after TACE and/or RFA between the Prestudy and Study Period

During the study period, a significantly lower number of cases were prescribed antibiotics compared with the prestudy period for patients undergoing TACE and/or RFA (11.2% versus 21.8%, *P* < 0.001). There was no difference in the incidence of infectious complications between the prestudy and study period. The average duration of antibiotics prescribed and length of hospital stay were also significantly lower during the study period (*P* < 0.001 and *P* < 0.001, resp.) (Table 4).

## 4. Discussion

This study showed the usefulness of PCT for making a decision regarding administration of antibiotics for patients who develop a fever after undergoing TACE and/or RFA for HCC. The accuracy of PCT measured on day 0 of fever was significantly higher, with an AUC value of 0.715, than that of other inflammatory markers such as CRP and WBC counts to discriminate a fever related to bacterial infection from a noninfectious fever. In addition, consecutively measured PCT levels on days 0, 1, 3, and 5 of fever were significantly higher in patients with bacterial infection than in those without infection. In our study, we used PCT level as a guide for determining whether or not to start antibiotic therapy. When we compared the total amount and duration of antibiotic prescriptions in patients with fever after TACE and/or RFA between the prestudy and study period, we found that a significantly lower number of patients were prescribed antibiotics in the study period than in the prestudy period. The average duration of antibiotic use and length of hospital stay were also decreased in the study period. There was no difference in the incidence of infectious complications between the prestudy and study period.

TACE and RFA are generally well-tolerated procedures for treating HCC. However, a considerable number of patients experience the postembolization or postablation syndrome, which is a transient self-limiting complex of symptoms or signs of fever and general malaise [4, 22]. In our study, fever after TACE and/or RFA was observed in 17.2% of patients, which is comparable to another study [13]. Even though the actual incidence of bacterial infection in patients undergoing TACE and/or RFA is quite low (0.8–2.5%) [4, 13], fever always causes a concern for infectious complications frequently leading physicians to prescribe unnecessary antibiotics.

We had five cases of hepatobiliary or gastrointestinal (GI) infections including primary *E. coli* bacteremia, 2 cases of UTI, and 2 cases of pneumonia. However, it is hard to exclude pneumonia and UTI that developed within 7 days from procedure-related infections. Moreover, five cases of hepatobiliary or GI infections also cannot be concluded as procedure related, because such infections are commonly occurring in patients with liver cirrhosis and/or HCC without TACE/RFA. For these reasons, we included all cases with bacterial infections developed within 7 days after procedure. The overall rate for infectious complication was 1.6% (9/563 cases), which is comparable to previous study [4].

In previous studies, PCT has been shown to be useful as a diagnostic marker for bacterial infection in patients with cirrhosis [23, 24]. However, little is known about the role of PCT in patients with fever after TACE and/or RFA [13]. In our study, the accuracy of PCT measured on the day of fever onset was significantly higher, with an AUC value of 0.715, than that of other inflammatory markers such as CRP (AUC 0.598) and WBC counts (AUC 0.502) to discriminate fever related to bacterial infection from noninfectious fever. This finding is in accord with a previous study of Dabbous et al., which showed superior diagnostic value of PCT over CRP in 42 patients with fever after TACE [13]. As the most widely used cutoff value of PCT is 0.5 ng/mL for the diagnosis of bacteremia or other invasive bacterial infection [16, 17, 25], we evaluated the predictive ability of PCT based on this cutoff value. The negative predictive value of a PCT level of <0.5 ng/mL on day 0 of fever was 90%. If the peak PCT value of <0.5 ng/mL was used as the cutoff value, the negative predictive value was 97.5%.

In this study, antibiotics were prescribed only when PCT was ≥0.5 ng/mL or a suspicious infection focus was present. As antibiotics had been empirically prescribed in patients with fever after TACE and/or RFA before the study period in our institute, we compared the total amount and duration of antibiotic use in patients with fever after TACE and/or RFA between the prestudy and study period. We found that a significantly lower number of patients were prescribed antibiotics in the study period than in the prestudy period. The average duration of antibiotic use was also decreased in the study period. There was no difference in the incidence of infectious complications between the prestudy and study period. Many institutes still prescribe antibiotics routinely to patients with fever after TACE or RFA [22, 26]. Some even recommend the use of prophylactic antibiotics despite a low incidence of infectious complications [27], probably because infections in patients with impaired liver function often lead to fatal outcomes. However, this excessive antibiotic use also leads to the emergence of antibiotic resistance, increased medical costs, and adverse effects of antibiotics. In our study, we showed the potential to reduce unnecessary antibiotic use with PCT level introduced as a guide to determining whether or not to start antibiotics.

This study has some limitations. First, as this study was performed in a single center and included only nine cases of bacterial infection, further extensive research is warranted before applying these results generally. Second, a PCT level of <0.5 ng/mL did not have 100% negative predictive value, and two patients with bacterial infection showed a low PCT level (<0.5 ng/mL) during 7 days of follow-up after fever onset. However, even in these cases, the administration of empirical antibiotics was not delayed as other tests such as chest radiography, a physical examination, and a microbiologic study showed evidence of bacterial infection. As there is no biomarker with 100% sensitivity or specificity for the diagnosis of infection, a physician's careful evaluation and other laboratory and microbiological tests should always be conducted at the same time.

In conclusion, PCT might be a biomarker to detect infectious complications after TACE and/or RFA in HCC patients, if physical examination and laboratory and microbiological tests are warranted to detect infection. PCT-guided antibiotic treatment could contribute to reduce unnecessary use of antibiotics without worsening outcomes in patients with fever after TACE and/or RFA.

## Figures and Tables

**Table 1:** Baseline characteristics of patients with and without bacterial infection among patients with fever after TACE and/or RFA.

Characteristics	Patients with fever after TACE and/or RFA		
	With bacterial infection (*N* = 9)	Without bacterial infection (*N* = 88)	*P* value
Male	7 (78)	67 (76)	>0.999
Age^∗^	73 (61–78)	63 (57–72)	0.072
Comorbidities			
Diabetes	4 (44)	32 (36)	0.723
Chronic lung disease	0 (0.0)	1 (1)	>0.999
Chronic kidney disease	0 (0.0)	13 (14.8)	0.603
Etiology of liver cirrhosis			
HBV	5 (56)	53 (61)	0.863
HCV	1 (11)	15 (17)	
Both HBV and HCV	0 (0)	2 (2)	
Alcohol	3 (33)	17 (19)	
Unknown etiology	0 (0)	1 (1)	
Child-Pugh score			
A	7 (78)	78 (89)	0.308
B	2 (22)	10 (11)	
C	0 (0)	0 (0)	
Ascites	3 (33)	10 (11)	0.099
Previous variceal bleeding	2 (22)	6 (7)	0.160
Previous hepatectomy	0 (0)	6 (7)	>0.999
TNM stage			
I	2 (22)	21 (24)	0.103
II	3 (33)	42 (48)	
IIIA	0 (0)	14 (16)	
IIIB	4 (44)	9 (10)	
IVA	0 (0)	1 (1)	
IVB	0 (0)	1 (1)	
Number of tumor			
Solitary	2 (22)	24 (27)	>0.999
Multiple	7 (78)	64 (73)	
Intervention			
TACE	7 (78)	59 (67)	0.847
RFA	1 (11)	21 (24)	
Sequential TACE and RFA	1 (11)	8 (9)	
Total dose of injected Adriamycin (mg)^∗^	20.0 (20–20)^†^	20.0 (20–20)^‡^	0.780
TDD	26.5 (25–30)^†^	26.0 (25–30)^‡^	0.911

^∗^Expressed as median (interquartile range); ^†^8 patients of TACE; ^‡^68 patients. TACE: transarterial chemoembolization; RFA: radiofrequency ablation; TDD: total dose delivered of TACE (Adriamycin (mg) + dose of iodized oil (mL)); HBV: hepatitis B virus; HCV: hepatitis C virus.

**Table 2:** Clinical characteristics of 9 patients with bacterial infection within 7 days after TACE and/or RFA.

Number	Gender	Age	Treatment	Diagnosis of infection	Bacterial etiology	Days of infection detected after TACE and/or RFA	PCT on day of fever	Peak PCT within 7 days	Outcome of infection
1	M	57	TACE	Cholecystitis	Bile, *E. hirae*	4	2.58	2.58	Cured
2	M	83	TACE	Pneumonia	—	5	0.72	0.72	Cured
3	M	55	TACE	UTI	—	4	0.23	2.40	Cured
4	M	75	TACE	Bacteremia, primary	Blood, *E. coli*	2	1.55	1.70	Cured
5	M	79	TACE	UTI	—	3	1.19	1.19	Cured
6	M	73	TACE	Pneumonia	Sputum, *E. coli*	7	0.21	0.24	Cured
7	F	77	Sequential TACE/RFA	Liver abscess	Blood, *E. casseliflavus*	7	0.16	0.59	Transferred to another hospital
8	M	71	TACE	Bacteremia, cholangitis	Blood, *E. gallinarum*	1	0.15	0.55	Cured
9	F	65	RFA	Bacteremia, RFA site cutaneous abscess	Blood, *S. aureus*	1	0.08	0.17	Cured

TACE: transarterial chemoembolization; RFA: radiofrequency ablation; UTI: urinary tract infection; PCT: procalcitonin.

**Table 3:** Comparison of laboratory data between patients with and without bacterial infection among patients who had fever after TACE and/or RFA.

Laboratory marker	Patients with fever after TACE and/or RFA		
	With bacterial infection (*N* = 9)	Without bacterial infection (*N* = 88)	*P* value
White blood cells (mm^3^), median (IQR)	8600 (6750–11,750)	8550 (6725–11,000)	0.985
Neutrophil (%)	78.9 (72.7–86.6)	73.6 (67.3–79.6)	0.147
C-reactive protein (mg/dL)	3.0 (0.5–15.6)	1.9 (0.5–5.1)	0.332
Procalcitonin (ng/mL)	0.2 (0.2–1.4)	0.1 (0.1–0.3)	0.035
T-bilirubin (mg/dL)	1.4 (1.3–2.4)	1.0 (0.8–1.5)	0.009
Alkaline phosphatase (U/L)	132.0 (118.5–178.0)	102.5 (82.5–135.8)	0.038
AST (U/L)	270.0 (148.5–469.0)	189.5 (97.8–319.3)	0.198
ALT (U/L)	183.0 (51.5–335.0)	156.5 (63.5–247.5)	0.580
Prothrombin time (INR)	1.2 (1.1–1.3)	1.1 (1.1–1.2)	0.134
Albumin (g/dL)	2.5 (3.1–4.1)	3.7 (3.3–4.0)	0.881

TACE: transarterial chemoembolization; RFA: radiofrequency ablation; IQR: interquartile range; INR: international normalized ratio; AST: aspartate transaminase; ALT: alanine transaminase.

**Table 4:** Comparison of antibiotics use in patients with fever after TACE and/or RFA between prestudy and study period.

	Study period *N* = 563	Prestudy period *N* = 569	*P*
TACE/RFA			0.041
TACE	433 (76.9)	416 (73.1)	
RFA	103 (18.3)	135 (23.7)	
Sequential TACE/RFA	27 (4.8)	18 (3.2)	
Proven bacterial infection	15 (2.7)	14 (2.5)	0.853
Proven bacterial infection within 7days	10 (1.8)	11 (1.9)	>0.9
Number of cases antibiotics prescribed	63 (11.2)	124 (21.8)	<0.001
Duration of antibiotics prescribed, median days (IQR)	6 (3–10)	7 (5–13)	<0.001
Length of hospital stay (after TACE/RFA), median days (IQR)	3 (2–4)	3 (2–6)	<0.001

TACE: transarterial chemoembolization; RFA: radiofrequency ablation; IQR: interquartile range.
